# Association of serum 25-hydroxyvitamins D_2_ and D_3_ with hearing loss in US adults: analysis from National Health and Nutrition Examination Survey, 2015–2016

**DOI:** 10.3389/fnut.2024.1390953

**Published:** 2024-07-26

**Authors:** Feng Chen, Yufan Gao, Yukai Wang, Ziyu Pan, Yinuo Chen, Huixiang Sheng, Qi Chen, Fan Ye

**Affiliations:** ^1^The Second School of Medicine, Wenzhou Medical University, Wenzhou, China; ^2^Department of Otorhinolaryngology, The First Affiliated Hospital of Wenzhou Medical University, Wenzhou, China; ^3^The School of Medicine, Zhejiang University, Hangzhou, China

**Keywords:** hearing loss, 25-hydroxyvitamin D_3_, 25-hydroxyvitamin D_2_, L-shaped association, dose–response

## Abstract

**Background:**

Hearing loss (HL) is increasingly recognized as a significant global public health issue, and research on its relationship with vitamin D levels has gained wider attention. However, the association between serum biomarkers 25-hydroxyvitamin D_2_ (25(OH)D_2_) and D_3_ (25(OH)D_3_) with different types of HL remains unclear. This study aimed to investigate the potential association of serum 25(OH)D_2_ and 25(OH)D_3_ with HL in US adults.

**Methods:**

A sample of 3,684 individuals aged 20–69 years from the 2015–2016 National Health and Nutrition Examination (NHANES) was analyzed in this study. HL was defined as a pure tone average > 25 dB in either ear at low frequencies (500, 1,000, 2000 Hz), speech frequencies (500, 1,000, 2000, 4,000 Hz), and high frequencies (3,000, 4,000, 6,000, 8,000 Hz). Logistic regression was employed to examine the association between serum 25(OH)D_2_ and 25(OH)D_3_ and HL. The study population was then stratified by age, gender, race, and education level to analyze potential differences between adults in different subgroups.

**Results:**

In the multivariate analysis, it was found that serum 25(OH)D_2_ was independently associated with low-frequency hearing loss (LFHL) (OR: 1.012 [95% CI, 1.005–1.020]) and speech-frequency hearing loss (SFHL) (OR: 1.011 [95% CI, 1.003–1.018]). Restrictive cubic spline analysis demonstrated a linear dose–response relationship between serum 25(OH)D_2_ levels and LFHL (*p* for linearity <0.001), as well as SFHL (*p* for linearity = 0.001). Conversely, an L-shaped association was observed between serum 25(OH)D_3_ levels and both LFHL (*p* for nonlinearity = 0.014) and SFHL (*p* for nonlinearity = 0.025), with threshold values identified at 35.3 and 36.5 nmol/L, respectively. Higher levels of serum 25(OH)D_3_ were associated with a lower probability of high-frequency hearing loss (HFHL) (OR: 0.994 [95% CI, 0.989–0.999]), with a threshold value identified at 53.9 nmol/L. Furthermore, a significant interaction between diabetes and serum 25(OH)D_2_ in LFHL was revealed through subgroup analysis (*p* = 0.041). In the non-diabetic population, serum 25(OH)D_2_ maintained its association with LFHL.

**Conclusion:**

Our findings suggested a positive association between serum 25(OH)D_2_ concentrations and both LFHL and SFHL in the studied cohort. Additionally, an L-shaped relationship was found between serum 25(OH)D_3_ and LFHL and SFHL, and higher levels of serum 25(OH)D_3_ were identified to be associated with a lower risk of HFHL.

## 1 Introduction

Hearing loss (HL) is increasingly recognized as an important public health issue in the contemporary age, affecting more than 5% of the world’s population (1). Multiple reasons have been attributed to the escalating prevalence of HL, including an aging population, noise from occupational or recreational settings, and the widespread use of headphone devices (2). HL not only hinders interpersonal interactions, but also exerts a profound impact on personal quality of life, and daily functioning (3–5), posing enormous health and economic burdens. Therefore, it is of great necessity to identify potential risk factors for potential HL.

The role of vitamin D in auditory impairment has increasingly become a focal point of research. Studies have revealed (6) that a deficiency in vitamin D can lead to the demineralization of cochlear calcium and disrupt microcirculation, which in turn contributes to changes in cochlear morphology and the incidence of HL. Further experimental investigations (7) have confirmed that vitamin D plays a critical role in the proliferation and differentiation of neural stem and progenitor cells, which is essential for preserving the normal function of the auditory nerve. In animal experiments, zebrafish embryos deficient in vitamin D receptor (VDR) gene were found to produce fewer sensory hair cells in their ears, resulting in HL and motor balance disorders (8). Similarly, mice lacking the VDR gene exhibited hypomineralization in their auditory ossicles, impairing sound transmission through the middle ear (9). Recent research has also demonstrated that VDR gene regulates brain natriuretic peptide via the cGMP-PKG signaling pathway, promoting neurite outgrowth and survival of cochlear spiral ganglion neurons (10). This growing body of evidence underscores the significance of vitamin D in maintaining auditory health.

In epidemiological investigations, to date, only a handful of studies have linked vitamin D with HL (11–14). A cross-sectional study revealed a relationship between vitamin D deficiency and an increased likelihood of low-frequency hearing loss (LFHL) and speech-frequency hearing loss (SFHL) among individuals aged 70 and over (13). Another study, based on data from the UK Biobank, found that vitamin D intake is negatively correlated with the incidence of HL (15). Several other research concentrated on the relationship between vitamin D and HL in specific populations; for example, vitamin D deficiency has been deemed as a risk factor for HL in patients with diabetes (11). However, more recent studies indicate that diabetes is correlated with high-frequency hearing loss (HFHL) (16). Therefore, since vitamin D deficiency is prevalent in diabetic patients (17, 18), HL may not be directly caused by diabetes but could be due to vitamin D deficiency in type 2 diabetes (T2DM) patients. This indicates that the current evidence is not certain and requires further investigation.

Given the backdrop of prior studies, it appears apparent that research revolving around the correlations between vitamin D and HL has primarily focused on the elderly or specific populations such as diabetics. Furthermore, the majority of studies have only focused on serum 25-hydroxyvitamin D (25(OH)D) or dietary vitamin D intake concerning HL, yet the individual contributions of serum 25-hydroxyvitamin D_3_ (25(OH)D_3_) and D_2_ (25(OH)D_2_) have been less studied. Since serum 25(OH)D is composed of both 25(OH)D_3_ and 25(OH)D_2_, it is important to distinguish between the two. Vitamin D_3_ (cholecalciferol) is primarily synthesized in the skin through sunlight exposure, whereas vitamin D_2_ (ergocalciferol) is mainly obtained from plant-based foods and supplements. Both forms undergo hydroxylation in the liver to form 25(OH)D_3_ and 25(OH)D_2_, respectively, which are the main circulating forms. Currently, serum 25(OH)D or 25(OH)D_3_ are the primary methods for assessing vitamin D status. Measuring 25(OH)D_2_ can provide a more comprehensive assessment of vitamin D status (19). With the use of high-performance liquid chromatography–tandem mass spectrometry (HPLC-MS/MS), 25(OH)D_2_ has further refined the classification of these measurements.

Therefore, the current study aims to clarify the association between serum 25(OH)D_2_ and 25(OH)D_3_ and HL separately, as well as to identify their optimal concentrations for potential clinical reference values.

## 2 Methods

### 2.1 Study participants

National Health and Nutrition Examination Survey (NHANES) (20) is a national, cross-sectional survey that collects health-related data from the US population every 2 years. The program is well representative undergoing a 4-stage rigid design and includes data derived from in-depth interviews, series of physical examinations, and laboratory test results. Detailed information on the study design, methodology, and data collection can be found on the official NHANES website.

**Figure 1:**
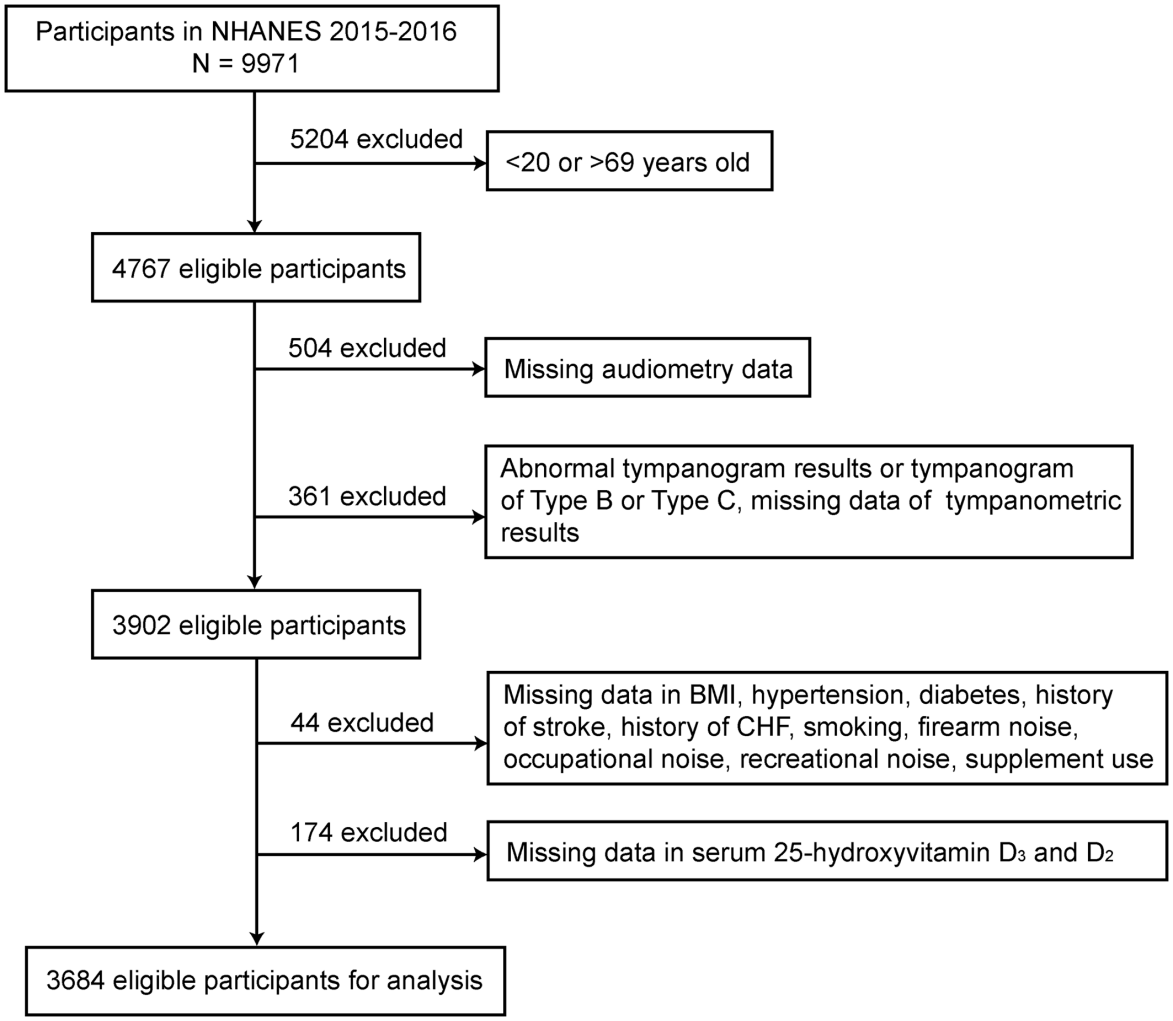
The flowchart of the participants’ selection process.

The current study utilized data of participants aged 20–69 years in NHANES from 2015 to 2016. Individuals were excluded if (1) audiometry data (*N* = 504) or abnormal tympanogram results, as well as those with type B and C tympanogram results were unavailable (21, 22) (*N* = 361); (2) data of other covariates such as body mass index (BMI), hypertension, diabetes, history of stroke, history of congestive heart failure, smoking, firearms noise exposure, occupational noise exposure, recreational noise exposure, and supplement use were missing (*N* = 44); (3) without serum vitamin D level data (*N* = 174). A total of 3,684 eligible participants remained for final analysis. The participant screening flowchart is presented in Figure 1.

### 2.2 Vitamin D levels

The NHANES 2015–2016 cycle used a HPLC-MS/MS method to measure serum 25(OH)D_2_ and serum 25(OH)D_3_ levels. When the 25(OH)D_2_ concentration is below the lower limit of detection, NHANES proposes using the lowest 25(OH)D_2_ concentration (1.45 nmol/L) as the input value (23, 24). For specific operations and details, refer to the NHANES Assessment of Vitamin D Levels document.

### 2.3 Audiometry

The audiometric requirements were met by all adults aged 20–69 years (25). The audiometric test was conducted by trained examiners in a soundproof room at the Mobile Examination Center (MEC) using an Interacoustics model AD226 audiometer with standard TDH-49P headphones and Etymotic EarTone 3A plug-in headphones to measure pure-tone air-conduction audiometry in both ears. Middle ear testing was performed using the Interacoustics Titan. Detailed instructions for this procedure can be found on the website (26).

HL was categorized as low-frequency, speech frequency, or high-frequency: LFHL defined as a mean of pure tones greater than 25 dB measured in either ear at 500, 1000, and 2000 Hz; SFHL defined as a mean of pure tones greater than 25 dB measured in either ear at 500, 1000, 2000, and 4,000 Hz; HFHL defined as a mean of pure tones greater than 25 dB measured in either ear at 3000, 4000, 6,000, and 8,000 Hz (13).

### 2.4 Covariates

Building upon previous research (25, 27, 28), we included the following covariates in our analysis: age, sex, race, level of education, BMI, hypertension, diabetes, history of stroke, history of congestive heart failure, smoking status, firearm noise, occupational noise, recreational noise, and supplement use. Definitions for medical histories including hypertension, diabetes, stroke, congestive heart failure, smoking status, and supplement use were all acquired through self-reports. Considering the potential impact of noise exposure on hearing, our analysis utilized three different sources of noise exposure, with definitions aligning with those found in prior literature (29).

### 2.5 Statistical analysis

Continuous variables were described as mean ± standard deviation or median (interquartile range) after Kolmogorov–Smirnov test for normality, and categorical variables as percentages. Depending on the characteristics of the variables, group differences were compared using the T-test, non-parametric tests, or chi-square tests. Multivariable logistic regression was employed to assess the relationship between serum 25(OH)D_2_, D_3_, and HL, followed by Generalized additive models (GAM) to further examine the nonlinear relationship. GAM provide flexibility by not assuming a predefined form for the relationship between the predictor and outcome variables. If a nonlinear relationship was detected, we applied piecewise logistic regression to identify threshold effects. This method involves dividing the predictor variable into segments and fitting separate logistic regression models within each segment. Restricted cubic splines allow for flexible modeling of nonlinear relationships by using piecewise polynomial functions that are smooth at the points where they join (knots). We visualized the relationship between serum 25(OH)D_2_ and HL using restricted cubic splines with three knots; and for the relationship between serum 25(OH)D_3_ and HL, five knots were used to further demonstrate an L-shaped relationship. Stratified logistic regression models were used for subgroup analyses, and likelihood ratio tests were applied to assess the effects and interactions within subgroups. All statistical analyses were performed using R version 4.3.0 (30) and EmpowerStats (31) software. A two-sided *p*-value <0.05 was considered statistically significant.

## 3 Results

### 3.1 Baseline characteristics of population with hearing loss

Among 3,684 participants aged 20–69 years, 328 had LFHL, 496 had SFHL, and 1,276 had HFHL. Those with older age, gender of male, Mexican-American ethnicity, lower education level, higher BMI, hypertension, diabetes, history of congestive heart failure, history of stroke, smoking, supplement use, occupational and recreational noise exposure were more likely to display HL in all three types (*p* < 0.05). Firearm noise exposure was found to be only associated with SFHL and HFHL (*p* < 0.05).

Across three types of HL, vitamin D levels showed different characteristics. Those with serum 25(OH)D_2_ concentration greater than 1.45 nmol/L were more inclined to be LFHL (*p* < 0.05), whereas serum 25(OH)D_3_ concentration was higher in SFHL and HFHL group (both *p* < 0.05) (Table 1).

**Table 1:** Characteristics of participants classified by hearing loss.

Variables	Total (*n* = 3684)	Low-frequency HL (*n* = 328)			Speech-frequency HL (*n* = 496)			High-frequency HL (*n* = 1276)		
		Yes	No	*p*-value	Yes	No	*p*-value	Yes	No	*p*-value
Age, years	43.81 ± 14.10	56.05 ± 11.29	42.61 ± 13.78	<0.001	56.71 ± 10.54	41.80 ± 13.51	<0.001	54.69 ± 10.94	38.05 ± 12.04	<0.001
Male, %	46.63	51.83	46.13	0.048	60.89	44.42	<0.001	58.39	40.41	<0.001
Race, %				0.001			<0.001			<0.001
Mexican American	18.84	23.17	18.41		22.38	18.29		20.53	17.94	
Other Hispanic	13.82	19.51	13.26		18.15	13.14		15.67	12.83	
Non-Hispanic White	29.23	25.61	29.59		29.84	29.14		32.29	27.62	
Non-Hispanic Black	21.63	17.07	22.08		14.31	22.77		18.26	23.42	
Other races	16.48	14.63	16.66		15.32	16.66		13.24	18.19	
Education level, %				<0.001			<0.001			<0.001
Less than 9th grade	9.72	17.99	8.91		17.54	8.50		14.42	7.23	
9-11th grade	11.45	15.85	11.03		15.73	10.79		14.73	9.72	
High school graduate or GED	21.44	19.51	21.63		21.37	21.46		22.73	20.76	
Some college or AA	30.92	28.35	31.17		27.22	31.49		28.13	32.39	
College graduate or more	26.47	18.29	27.26		18.15	27.76		19.98	29.90	
BMI, kg/m^2^	29.81 ± 7.37	30.81 ± 7.17	29.71 ± 7.38	0.010	30.78 ± 7.14	29.66 ± 7.39	0.002	30.45 ± 6.91	29.47 ± 7.57	<0.001
Hypertension, %	30.24	50.00	28.31	<0.001	48.99	27.32	<0.001	45.45	22.18	<0.001
Diabetes, %	13.22	27.13	11.86	<0.001	30.44	10.54	<0.001	23.75	7.64	<0.001
History of CHF, %	1.82	6.10	1.40	<0.001	5.85	1.19	<0.001	3.61	0.87	<0.001
History of stroke, %	2.47	7.01	2.03	<0.001	6.85	1.79	<0.001	5.17	1.04	<0.001
Smoking, %	39.93	49.09	39.03	<0.001	51.61	38.11	<0.001	50.78	34.18	<0.001
Supplement use, %	49.35	54.88	48.81	0.036	55.85	48.34	0.002	53.68	47.05	<0.001
Firearm noise, %	38.14	38.11	38.14	0.991	42.74	37.42	0.023	42.01	36.09	<0.001
Occupational noise, %	32.49	38.41	31.91	0.016	41.53	31.09	<0.001	40.44	28.28	<0.001
Recreational noise, %	14.09	17.68	13.74	0.050	19.35	13.27	<0.001	16.69	12.71	0.001
25(OH)D_2_ >1.45 nmol/L, %	18.59	22.87	18.18	0.037	19.15	18.51	0.731	18.26	18.77	0.705
25(OH)D_3_, nmol/L	55.20 (39.68–72.80)	57.90 (41.38–73.73)	55.00 (39.48–72.70)	0.235	57.70 (42.08–75.45)	54.80 (39.10–72.43)	0.018	59.00 (42.90–75.80)	53.20 (37.88–71.50)	<0.001

HL, hearing loss; GED, General Educational Development; AA, Associate in Arts; CHF, congestive heart failure; BMI, body mass index; 25(OH)D_2_, 25-hydroxyvitamin D_2_; 25(OH)D_3_, 25-hydroxyvitamin D_3_.

### 3.2 Multivariable logistic regression

Multivariable binary logistic regression was used to explore the association between serum 25(OH)D_2_, D_3_, and HL. Crude Model 1 adjusted for demographic variables including age, gender, race, education level and Model 2 fully adjusted for all potential covariates described above in Method (Table 2). In Model 1, serum 25(OH)D_2_ concentration was associated with LFHL (OR:1.013 [95% CI, 1.005–1.020], *p* < 0.001) and SFHL (OR:1.012 [95% CI, 1.005–1.020], *p* = 0.001), but no significance was identified in HFHL (OR:1.003 [95% CI, 0.995–1.011], *p* = 0.438). Serum 25(OH)D_3_ concentration was associated with SFHL (OR:0.996 [95% CI, 0.992–1.000], *p* = 0.049) and HFHL (OR:0.996 [95% CI, 0.993–0.999], *p* = 0.023), but no significance found in LFHL (OR:0.995 [95% CI, 0.991–1.000], *p* = 0.057). To further enhance the robustness of the association between serum 25(OH)D_3_ and 25(OH)D_2_ levels and HL in adults, we additionally adjusted for confounding factors such as serum calcium, serum phosphorus, and a history of renal insufficiency (Supplementary Table 3). Sensitivity analyses demonstrated that the association between serum 25(OH)D_3_ and 25(OH)D_2_ levels and HL in adults remained significant, indicating a strong robustness of our findings.

**Table 2:** Multivariate logistic regression models assessing the relationship between 25-hydroxyvitamin D_2_, D_3_ and hearing loss.

Classifications			25(OH)D_2_	25(OH)D_3_
Low-frequency HL	Model 1	OR (95% CI)	1.013 (1.005–1.020)	0.995 (0.991–1.000)
		*p*-value	< 0.001	0.057
	Model 2	OR (95% CI)	1.012 (1.005–1.020)	0.997 (0.992–1.001)
		*p*-value	< 0.001	0.171
Speech-frequency HL	Model 1	OR (95% CI)	1.012 (1.005–1.020)	0.996 (0.992–1.000)
		*p*-value	0.001	0.049
	Model 2	OR (95% CI)	1.011 (1.003–1.018)	0.997 (0.993–1.002)
		*p*-value	0.004	0.198
High-frequency HL	Model 1	OR (95% CI)	1.003 (0.995–1.011)	0.996 (0.993–0.999)
		*p*-value	0.438	0.023
	Model 2	OR (95% CI)	1.002 (0.994–1.009)	0.997 (0.994–1.001)
		*p*-value	0.693	0.147

Model 1: adjusted for age, gender, race, education.

Model 2: adjusted for age, gender, race, education, BMI, hypertension, diabetes, history of CHF, history of stroke, smoking, supplement use, firearm noise, occupational noise, recreational noise.

25(OH)D_2_, 25-hydroxyvitamin D_2_; 25(OH)D_3_, 25-hydroxyvitamin D_3_; HL, hearing loss; OR, odd ratio; CI, confidence interval.

In Model 2, serum 25(OH)D_2_ concentration remained consistent results compared to Model 1. It was found to be associated with LFHL (OR:1.012 [95% CI, 1.005–1.020], *p* < 0.001) and SFHL (OR:1.011 [95% CI, 1.003–1.018], *p* = 0.004), but not in HFHL (OR:1.002 [95% CI, 0.994–1.009], *p* = 0.693). Nevertheless, there was no significance reported for the association between serum 25(OH)D_3_ concentration and LFHL (OR:0.997 [95% CI, 0.992–1.001], *p* = 0.171), SFHL (OR:0.997 [95% CI, 0.993–1.002], *p* = 0.198), or HFHL (OR:0.997 [95% CI, 0.994–1.001], *p* = 0.147).

### 3.3 Threshold effects and restricted cubic splines

**Figure 2:**
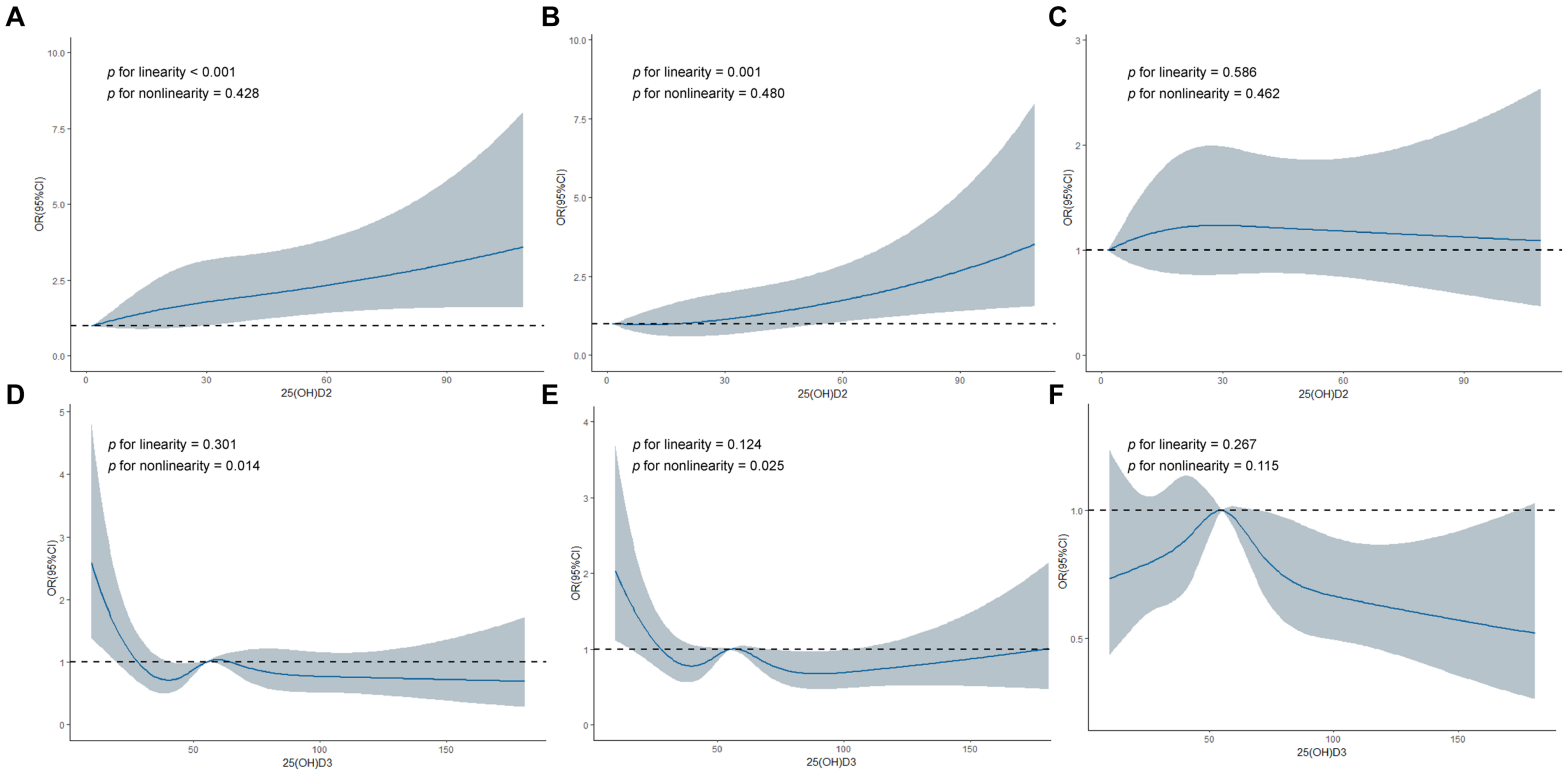
Restricted cubic spline regression analyses of 25-hydroxyvitamin D_2_, D_3_ and hearing loss. **(A)** Association between 25(OH)D_2_ and Low-frequency HL. **(B)** Association between 25(OH)D_2_ and Speech-frequency HL. **(C)** Association between 25(OH)D_2_ and High-frequency HL. **(D)** Association between 25(OH)D_3_ and Low-frequency HL. **(E)** Association between 25(OH)D_3_ and Speech-frequency HL. **(F)** Association between 25(OH)D_3_ and High-frequency HL. 25(OH)D_2_, 25-hydroxyvitamin D_2_; 25(OH)D_3_, 25-hydroxyvitamin D_3_; HL, hearing loss; OR, odd ratio; CI, confidence interval.

To further explore whether a nonlinear relationship existed between serum 25(OH)D_2_, D_3_, and HL, we implemented piecewise logistic regression using GAM and recursive methods to calculate effect thresholds. Restricted cubic splines were then used to visualize the relationship. We found a linear dose–response relationship between serum 25(OH)D_2_ and LFHL (*p* for linearity <0.001) and SFHL (*p* for linearity = 0.001). Comparatively, serum 25(OH)D_3_ had an L-shaped relationship with LFHL (*p* for nonlinearity = 0.014) and SFHL (*p* for nonlinearity = 0.025), with critical values of 35.3 and 36.5 nmol/L, respectively (Figure 2 and Table 3).

**Table 3:** The results of two-piecewise logistic regression model.

A. Associations of 25-hydroxyvitamin D_2_ with hearing loss						
Classifications	Low-frequency HL		Speech-frequency HL		High-frequency HL	
	OR (95% CI)	*p*-value	OR (95% CI)	*p*-value	OR (95% CI)	*p*-value
Fitting model by standard regression	1.012 (1.005–1.020)	0.001	1.011 (1.003–1.018)	0.004	1.002 (0.994–1.009)	0.693
**Fitting model by two-piecewise regression**						
Inflection point of 25(OH)D_2_ (K)	2.41		8.40		6.58	
<K	1.144 (0.798–1.639)	0.464	0.987 (0.919–1.059)	0.710	1.037 (0.965–1.115)	0.323
≥K	1.011 (1.003–1.019)	0.008	1.013 (1.003–1.023)	0.010	0.999 (0.990–1.008)	0.808
*p* for log likelihood ratio test		0.509		0.500		0.342

B. Associations of 25-hydroxyvitamin D_3_ with hearing loss						
Classifications	Low-frequency HL		Speech-frequency HL		High-frequency HL	
	OR (95% CI)	*p*-value	OR (95% CI)	*p*-value	OR (95% CI)	*p*-value
Fitting model by standard regression	0.997 (0.992–1.001)	0.171	0.997 (0.993–1.002)	0.198	0.997 (0.994–1.001)	0.147
**Fitting model by two-piecewise regression**						
Inflection point of 25(OH)D_3_ (K)	35.3		36.5		53.9	
<K	0.963 (0.938–0.988)	0.004	0.972 (0.950–0.995)	0.017	1.007 (0.997–1.016)	0.169
≥K	1.000 (0.994–1.005)	0.961	1.000 (0.995–1.004)	0.874	0.994 (0.989–0.999)	0.013
*p* for log likelihood ratio test		0.010		0.033		0.036

25(OH)D_2_, 25-hydroxyvitamin D_2_; 25(OH)D_3_, 25-hydroxyvitamin D_3_; HL, hearing loss; OR, odd ratio; CI, confidence interval.

Piecewise logistic regression showed that serum 25(OH)D_2_ concentration greater than 2.41 nmol/L was significantly associated with LFHL (OR:1.011 [95% CI, 1.003–1.019], *p* = 0.008), but there was no statistical difference compared to standard logistic regression (*p* for log likelihood ratio test = 0.509), indicating no threshold effect. Similarly, serum 25(OH)D_2_ concentration greater than 8.40 nmol/L was significantly associated with SFHL (OR:1.013 [95% CI, 1.003–1.023], *p* = 0.010), but there again was no threshold effect (*p* for log likelihood ratio test = 0.500). As for HFHL, neither standard regression nor piecewise logistic regression indicated any association between serum 25(OH)D_2_ and HFHL.

Serum 25(OH)D_3_ demonstrated threshold effects in all three types of HL (*p* for log likelihood ratio test <0.05). Specifically, lower levels (<35.3 nmol/L) of serum 25(OH)D_3_ were associated with a higher likelihood of LFHL (OR:0.963 [95% CI, 0.938–0.988], *p* = 0.004); lower levels (<36.5 nmol/L) of serum 25(OH)D_3_ were associated with a higher likelihood of SFHL (OR:0.972 [95% CI, 0.950–0.995], *p* = 0.017); higher levels (>53.9 nmol/L) of serum 25(OH)D_3_ were associated with a lower likelihood of HFHL (OR:0.994 [95% CI, 0.989–0.999], *p* = 0.013) (Table 3).

### 3.4 Subgroup analysis

Subgroup analyses were conducted incorporating covariates of interest, containing age, gender, race, education level, BMI, hypertension, diabetes, and smoking (Supplementary Tables 1, 2). The results revealed a significant interaction between diabetes and serum 25(OH)D_2_ in LFHL (*p* = 0.041). Serum 25(OH)D_2_ retained its association with LFHL in the non-diabetic population (OR:1.020 [95% CI, 1.010–1.031], *p* < 0.001). Moreover, serum 25(OH)D_2_ remained associated with LFHL in populations aged 37–69, females, Mexican-Americans, those with a high school education level, and those with a BMI > 30 (*p* < 0.05). Serum 25(OH)D_2_ was also associated with SFHL in populations aged 53–69, females, Mexican-Americans, those with less than a ninth-grade education level, with a BMI > 30, with hypertension, non-diabetics, and smokers (*p* < 0.05). Unfortunately, there present no significant association in HFHL across all subgroups (Supplementary Table 1).

When it comes to the part of serum 25(OH)D_3_, for Mexican-Americans and those with a high school education level, it was found to be associated with smaller likelihood of SFHL (*p* < 0.05). Moreover, in females and those with a BMI 25–30 subgroup, 25(OH)D_3_ was associated with smaller likelihood of HFHL (*p* < 0.05). No association between serum 25(OH)D_3_ and LFHL was displayed in the subgroups (Supplementary Table 2).

## 4 Discussion

Utilizing a considerable sample size from the NHANES 2015–2016 cycle, we investigated potential relationships between serum 25(OH)D_2_, D_3_ levels and HL in adults. Major findings suggested that serum 25(OH)D_2_ had a positive independent association with both LFHL and SFHL. Restricted cubic splines confirmed a linear dose–response relationship between serum 25(OH)D_2_ and these forms of HL. Interestingly, an L-shaped relationship was observed between serum 25(OH)D_3_ and both LFHL and SFHL, with critical values of 35.3 nmol/L and 36.5 nmol/L, respectively. Higher levels of serum 25(OH)D_3_ were associated with a lower likelihood of HFHL, with a critical value of 53.9 nmol/L. Subgroup analysis revealed a significant interaction between diabetes and serum 25(OH)D_2_ in LFHL outcomes, maintaining its significant association within the non-diabetic population.

Previous studies have primarily focused on the association between serum 25(OH)D concentration and HL (11, 12, 32, 33). One study (13) using a sample of 1,123 older adults from NHANES (2005–2010) suggested that low serum 25(OH)D concentration was positively correlated with the occurrence of LFHL and SFHL but not HFHL. This cross-sectional study’s findings align with our observation of an L-shaped relationship between serum 25(OH)D_3_ concentration and HL. Another NHANES study (14) also tried to establish a correlation between vitamin D deficiency and bilateral low-frequency hearing impairment and sensorineural HL. Other studies have revealed a negative correlation between serum 25(OH)D_3_ and depression, while serum 25(OH)D_2_ has an inverted U-shaped relationship with depression (34). Additionally, serum 25(OH)D and 25(OH)D3 concentrations are positively correlated with cognitive function (23), all linked to mental disorders.

The L-shaped relationship between serum 25(OH)D_3_ and HL could be explained by several mechanisms. To begin with, the presence of vitamin D deficiency might contribute to HL by affecting cochlear bone density (35), leading to cochlear demineralization and microcirculation changes, altering calcium metabolism, and causing morphological changes in the cochlea that eventually result in hearing decline (6). Another underlying mechanism could be that vitamin D can cross the blood–brain barrier (36) and distribute unevenly among various brain regions (37). This uneven distribution affects the proliferation and differentiation of neural stem cells and progenitors (7), leading to impaired neuronal proliferation and differentiation, affecting vestibuloauditory neural function and resulting in hearing decline if vitamin D is deficient.

The positive independent association identified between serum 25(OH)D_2_ and low and SFHL can be putatively explained by the interaction between vitamin D2, D3. Previous research (38, 39) has shown that vitamin D2 supplementation can decrease circulating concentrations of 25(OH)D_3_, potentially as a result of affecting the metabolism of vitamin D3. This might be partly explained by the competition between vitamin D2 and D3 for the same hydroxylation pathways, with vitamin D3 being a more effective factor in maintaining and elevating serum total 25(OH)D levels (40). In light of this, high levels of vitamin D2 might competitively affect the activation of vitamin D3, hindering the body’s ability to maintain adequate levels of total serum 25(OH)D, and ultimately lead to the occurrence of HL.

Subgroup analysis observed a significant interaction between diabetes and serum 25(OH)D_2_ in LFHL. Previous research (41) has shown that vitamin D are negatively associated with the risk of T2DM, potentially because vitamin D plays a underlying part in regulating the process of immune and inflammatory cells proliferation, differentiation and function, upregulating anti-inflammatory pathways and downregulating the activation of these cells, thereby reducing the risk of T2DM (42). Longitudinal studies have also correlated diabetes with an increased risk of moderate or severe HL (43).

This study has several strengths. Firstly, we separately investigated the associations between serum 25(OH)D_2_ and 25(OH)D_3_, and adult HL, uncovering contrasting trends between these markers. To our knowledge, this study is the first to explore the association in this perspective, as previous research was limited to seeking the association between total serum 25(OH)D and HL. Our research underscores the necessity of individually considering serum 25(OH)D_2_ and 25(OH)D_3_ when studying the correlation between vitamin D and HL. Secondly, the large sample size provided by NHANES ensured the reliability of our statistical results. Restricted cubic splines and threshold analysis assessed the L-shaped relationship between serum 25(OH)D_3_ and LFHL and SFHL. The primary limitation of this study lies in its cross-sectional design, which precludes the determination of causality. Additionally, due to database constraints, it was not possible to adjust for all potential confounding variables, such as osteoporosis and parathyroid hormone levels, which may influence vitamin D levels and auditory health. Moreover, when the 25(OH)D_2_ concentration is below the detection limit, we use the minimum 25(OH)D_2_ concentration (1.45 nmol/L) as the input value. This does not reflect the patient’s true 25(OH)D_2_ concentration. Assessing vitamin D levels based on a single test may be insufficient to fully capture its impact on HL, and further research is needed to determine the precise effects and implications of 25(OH)D_2_ at accurate concentrations. Based on our findings, which provide potential epidemiological evidence for differing vitamin D supplementation categories, further prospective research relating to this topic is encouraged to establish causality between these factors.

## 5 Conclusion

This study consolidated that in the adult population of the United States, serum 25(OH)D_2_ concentration was positively associated with LFHL and SFHL, while serum 25(OH)D_3_ exhibited an L-shaped relationship with LFHL and SFHL, with critical values of 35.3 and 36.5 nmol/L, respectively. Higher levels of serum 25(OH)D_3_ were associated with a lower likelihood of HFHL, with a critical value of 53.9 nmol/L. Furthermore, serum 25(OH)D_2_ retained its association with LFHL in the non-diabetic population.

Future research should focus on longitudinal and interventional studies to establish causality and explore the effects of vitamin D supplementation on HL. Mechanistic studies are needed to understand the biological processes involved, while studies on diverse populations can help determine the universal applicability of the findings. Comparative studies between vitamin D2 and D3, as well as genetic studies to explore individual responses, are also recommended.

## Data availability statement

Publicly available datasets were analyzed in this study. This data can be found here: https://www.cdc.gov/nchs/nhanes/index.htm.

## Ethics statement

The studies involving humans were approved by the ethics protocol was approved by the Research Ethics Review Board of National Center for Health Statistics (https://www.cdc.gov/nchs/nhanes/irba98.htm). The studies were conducted in accordance with the local legislation and institutional requirements. The participants provided their written informed consent to participate in this study.

## Author contributions

FC: Writing – original draft. YG: Writing – original draft. YW: Writing – original draft. ZP: Writing – original draft. YC: Writing – original draft. HS: Writing – original draft. QC: Writing – original draft. FY: Writing – review & editing.
