# Supplementary material for: Association of serum 25-hydroxyvitamins D2 and D3 with hearing loss in US adults: analysis from National Health and Nutrition Examination Survey, 2015–2016
Source: Front Nutr. 2024 Jul 26;11:1390953. doi: 10.3389/fnut.2024.1390953 (PMC11310169; doi:10.3389/fnut.2024.1390953)
Supplement: Supplementary file 1 [file Table_1.docx]

**SUPPLEMENTARY MATERIALS**

**Association of serum 25-hydroxyvitamins D_2_ and D_3_ with hearing loss in US adults: analysis from National Health and Nutrition Examination Survey (NHANES), 2015–2016**

**Short Title:** Vitamin D2, D3 in hearing loss

**Authors:**

Feng Chen^1§^, Yufan Gao^1§^, Yukai Wang^1§^, Ziyu Pan^1^, Yinuo Chen^1^, Huixiang Sheng^1^, Qi Chen^2^, Fan Ye^3^

**Institutions:**

^1^The Second School of Medicine, Wenzhou Medical University, Wenzhou, China

^2^The School of Medicine, Zhejiang University, Hangzhou, China

^3^Department of Otorhinolaryngology, The First Affiliated Hospital of Wenzhou Medical University, Wenzhou, China

**§Co-first author:** Feng Chen, Yufan Gao and Yukai Wang

**Corresponding author:** Fan Ye

Fan Ye, Department of Otorhinolaryngology, The First Affiliated Hospital of Wenzhou Medical University Shangcai Village, Wenzhou, Zhejiang 325000, China. E-mail: yfyfyfpp@163.com.

**Supplemental Tables**

**Supplementary Table 1.** Subgroup analysis of the relationship between 25-hydroxyvitamin D_2_ and hearing loss.

| Subgroups | N | **Low-frequency HL** | | | **Speech-frequency HL** | | | **High-frequency HL** | | |
| --- | --- | --- | --- | --- | --- | --- | --- | --- | --- | --- |
|  |  | *p*-value | OR (95% CI) | *p* interaction | *p*-value | OR (95% CI) | *p* interaction | *p*-value | OR (95% CI) | *p* interaction |
| Age, year |  |  |  | 0.394 |  |  | 0.594 |  |  | 0.500 |
| 20-36 | 1333 | 0.578 | 0.955 (0.812-1.123) |  | 0.645 | 0.970 (0.853-1.104) |  | 0.226 | 1.017 (0.990-1.045) |  |
| 37-52 | 1186 | 0.011 | 1.018 (1.004-1.032) |  | 0.232 | 1.009 (0.994-1.024) |  | 0.791 | 0.998 (0.985-1.011) |  |
| 53-69 | 1165 | 0.004 | 1.012 (1.004-1.021) |  | 0.004 | 1.013 (1.004-1.021) |  | 0.422 | 1.004 (0.994-1.013) |  |
| Gender (Male) |  |  |  | 0.364 |  |  | 0.286 |  |  | 0.551 |
| Yes | 1718 | 0.414 | 1.006 (0.991-1.022) |  | 0.586 | 1.004 (0.990-1.018) |  | 0.722 | 0.997 (0.981-1.013) |  |
| No | 1966 | < 0.001 | 1.014 (1.006-1.023) |  | 0.003 | 1.013 (1.004-1.021) |  | 0.539 | 1.003 (0.994-1.011) |  |
| Race |  |  |  | 0.758 |  |  | 0.798 |  |  | 0.844 |
| Mexican American | 694 | 0.016 | 1.019 (1.004-1.035) |  | 0.049 | 1.016 (1.000-1.032) |  | 0.371 | 1.008 (0.991-1.025) |  |
| Other Hispanic | 509 | 0.124 | 1.013 (0.996-1.031) |  | 0.410 | 1.007 (0.990-1.025) |  | 0.457 | 0.993 (0.976-1.011) |  |
| Non-Hispanic White | 1077 | 0.091 | 1.014 (0.998-1.031) |  | 0.095 | 1.014 (0.998-1.030) |  | 0.784 | 1.002 (0.986-1.018) |  |
| Non-Hispanic Black | 797 | 0.171 | 1.013 (0.995-1.026) |  | 0.122 | 1.013 (0.997-1.029) |  | 0.772 | 1.003 (0.985-1.020) |  |
| Other races | 607 | 0.727 | 1.003 (0.984-1.023) |  | 0.753 | 1.003 (0.986-1.020) |  | 0.936 | 1.001 (0.985-1.016) |  |
| Education level |  |  |  | 0.720 |  |  | 0.548 |  |  | 0.109 |
| Less than 9th grade | 358 | 0.118 | 1.013 (0.997-1.030) |  | 0.041 | 1.024 (1.001-1.048) |  | 0.397 | 1.010 (0.987-1.034) |  |
| 9-11th grade | 422 | 0.632 | 1.006 (0.982-1.030) |  | 0.293 | 1.016 (0.987-1.046) |  | 0.135 | 0.983 (0.960-1.005) |  |
| High school graduate or  GED | 790 | 0.012 | 1.021 (1.005-1.038) |  | 0.168 | 1.011 (0.996-1.026) |  | 0.150 | 1.017 (0.994-1.041) |  |
| Some college or AA | 1139 | 0.489 | 1.006 (0.990-1.022) |  | 0.804 | 1.002 (0.986-1.018) |  | 0.202 | 0.991 (0.977-1.005) |  |
| College graduate or  more | 975 | 0.069 | 1.013 (0.999-1.027) |  | 0.205 | 1.009 (0.995-1.023) |  | 0.450 | 1.005 (0.992-1.018) |  |
| BMI, kg/m^2^ |  |  |  | 0.492 |  |  | 0.623 |  |  | 0.992 |
| ≤18.5 | 56 | 0.079 | 1.131 (0.986-1.298) |  | 0.119 | 1.118 (0.972-1.287) |  | 0.805 | 1.020 (0.869-1.198) |  |
| 18.5-25 | 973 | 0.097 | 1.019 (0.997-1.041) |  | 0.556 | 1.006 (0.986-1.027) |  | 0.927 | 0.999 (0.978-1.020) |  |
| 25-30 | 1140 | 0.155 | 1.010 (0.996-1.023) |  | 0.111 | 1.011 (0.998-1.024) |  | 0.799 | 1.002 (0.988-1.015) |  |
| >30 | 1515 | 0.012 | 1.012 (1.003-1.022) |  | 0.020 | 1.011 (1.002-1.021) |  | 0.750 | 1.002 (0.992-1.011) |  |
| Hypertension |  |  |  | 0.646 |  |  | 0.750 |  |  | 0.412 |
| Yes | 1114 | 0.012 | 1.011 (1.002-1.020) |  | 0.011 | 1.011 (1.003-1.020) |  | 0.425 | 1.004 (0.994-1.014) |  |
| No | 2570 | 0.021 | 1.015 (1.002-1.028) |  | 0.181 | 1.009 (0.996-1.022) |  | 0.688 | 0.998 (0.985-1.010) |  |
| Diabetes |  |  |  | 0.041 |  |  | 0.330 |  |  | 0.421 |
| Yes | 487 | 0.414 | 1.005 (0.994-1.016) |  | 0.165 | 1.007 (0.997-1.017) |  | 0.741 | 0.998 (0.987-1.009) |  |
| No | 3197 | <0.001 | 1.020 (1.010-1.030) |  | 0.007 | 1.014 (1.004-1.025) |  | 0.409 | 1.004 (0.994-1.014) |  |
| Smoking |  |  |  | 0.600 |  |  | 0.572 |  |  | 0.851 |
| Yes | 1471 | 0.010 | 1.015 (1.004-1.026) |  | 0.022 | 1.013 (1.002-1.024) |  | 0.913 | 1.001 (0.989-1.012) |  |
| No | 2213 | 0.027 | 1.011 (1.001-1.021) |  | 0.069 | 1.009 (0.999-1.018) |  | 0.671 | 1.002 (0.993-1.012) |  |

**Abbreviations:** HL, hearing loss; GED, General Educational Development; AA, Associate in Arts; CHF, congestive heart failure; BMI, body mass index; OR, odd ratio; CI, confidence interval.

**Supplementary Table 2.** Subgroup analysis of the relationship between 25-hydroxyvitamin D_3_ and hearing loss.

| Subgroups | N | **Low-frequency HL** | | | **Speech-frequency HL** | | | **High-frequency HL** | | |
| --- | --- | --- | --- | --- | --- | --- | --- | --- | --- | --- |
|  |  | *p*-value | OR (95% CI) | *p* interaction | *p*-value | OR (95% CI) | *p* interaction | *p*-value | OR (95% CI) | *p* interaction |
| Age, year |  |  |  | 0.891 |  |  | 0.743 |  |  | 0.984 |
| 20-36 | 1333 | 0.863 | 1.001 (0.986-1.017) |  | 0.714 | 0.997 (0.981-1.013) |  | 0.746 | 0.998 (0.989-1.008) |  |
| 37-52 | 1186 | 0.646 | 0.997 (0.986-1.009) |  | 0.351 | 0.995 (0.986-1.005) |  | 0.737 | 0.999 (0.993-1.005) |  |
| 53-69 | 1165 | 0.349 | 0.997 (0.992-1.003) |  | 0.790 | 0.999 (0.995-1.004) |  | 0.768 | 0.999 (0.995-1.004) |  |
| Gender (Male) |  |  |  | 0.233 |  |  | 0.353 |  |  | 0.084 |
| Yes | 1718 | 0.949 | 1.000 (0.993-1.007) |  | 0.795 | 0.999 (0.993-1.005) |  | 0.715 | 1.001 (0.995-1.007) |  |
| No | 1966 | 0.076 | 0.994 (0.988-1.001) |  | 0.116 | 0.996 (0.990-1.001) |  | 0.032 | 0.995 (0.991-1.000) |  |
| Race |  |  |  | 0.803 |  |  | 0.506 |  |  | 0.520 |
| Mexican American | 694 | 0.096 | 0.990 (0.978-1.002) |  | 0.039 | 0.989 (0.978-0.999) |  | 0.073 | 0.992 (0.983-1.001) |  |
| Other Hispanic | 509 | 0.603 | 0.997 (0.986-1.008) |  | 0.661 | 0.998 (0.987-1.008) |  | 0.536 | 0.997 (0.987-1.007) |  |
| Non-Hispanic White | 1077 | 0.675 | 0.998 (0.990-1.007) |  | 0.630 | 0.998 (0.991-1.005) |  | 0.231 | 0.997 (0.991-1.002) |  |
| Non-Hispanic Black | 797 | 0.669 | 0.997 (0.985-1.009) |  | 0.836 | 0.999 (0.988-1.010) |  | 0.764 | 0.999 (0.991-1.007) |  |
| Other races | 607 | 0.782 | 0.999 (0.988-1.009) |  | 0.919 | 1.000 (0.991-1.010) |  | 0.555 | 1.003 (0.994-1.011) |  |
| Education level |  |  |  | 0.295 |  |  | 0.179 |  |  | 0.827 |
| Less than 9th grade | 358 | 0.827 | 0.999 (0.988-1.010) |  | 0.685 | 0.998 (0.987-1.008) |  | 0.114 | 0.992 (0.981-1.002) |  |
| 9-11th grade | 422 | 0.189 | 1.008 (0.996-1.021) |  | 0.231 | 1.007 (0.996-1.019) |  | 0.886 | 0.999 (0.989-1.010) |  |
| High school graduate or  GED | 790 | 0.100 | 0.991 (0.981-1.002) |  | 0.024 | 0.989 (0.980-0.999) |  | 0.361 | 0.996 (0.989-1.004) |  |
| Some college or AA | 1139 | 0.280 | 0.995 (0.987-1.004) |  | 0.299 | 0.996 (0.989-1.004) |  | 0.591 | 0.998 (0.992-1.004) |  |
| College graduate or  more | 975 | 0.270 | 0.995 (0.986-1.004) |  | 0.827 | 0.999 (0.991-1.007) |  | 0.514 | 0.998 (0.992-1.004) |  |
| BMI, kg/m^2^ |  |  |  | 0.586 |  |  | 0.598 |  |  | 0.055 |
| ≤18.5 | 56 | 0.358 | 0.969 (0.907-1.036) |  | 0.299 | 0.964 (0.901-1.033) |  | 0.073 | 0.972 (0.943-1.003) |  |
| 18.5-25 | 973 | 0.144 | 0.993 (0.984-1.002) |  | 0.224 | 0.995 (0.988-1.003) |  | 0.547 | 0.998 (0.992-1.004) |  |
| 25-30 | 1140 | 0.749 | 0.999 (0.991-1.006) |  | 0.532 | 0.998 (0.991-1.005) |  | 0.045 | 0.994 (0.988-1.000) |  |
| >30 | 1515 | 0.496 | 0.997 (0.990-1.005) |  | 0.712 | 0.999 (0.992-1.005) |  | 0.493 | 1.002 (0.996-1.008) |  |
| Hypertension |  |  |  | 0.297 |  |  | 0.940 |  |  | 0.372 |
| Yes | 1114 | 0.089 | 0.994 (0.988-1.001) |  | 0.301 | 0.997 (0.991-1.003) |  | 0.098 | 0.996 (0.990-1.001) |  |
| No | 2570 | 0.773 | 0.999 (0.992-1.006) |  | 0.371 | 0.997 (0.991-1.003) |  | 0.524 | 0.999 (0.994-1.003) |  |
| Diabetes |  |  |  | 0.725 |  |  | 0.921 |  |  | 0.710 |
| Yes | 487 | 0.600 | 0.998 (0.989-1.006) |  | 0.503 | 0.997 (0.990-1.005) |  | 0.326 | 0.996 (0.988-1.004) |  |
| No | 3197 | 0.174 | 0.996 (0.990-1.002) |  | 0.242 | 0.997 (0.992-1.002) |  | 0.221 | 0.998 (0.994-1.001) |  |
| Smoking |  |  |  | 0.348 |  |  | 0.363 |  |  | 0.621 |
| Yes | 1471 | 0.105 | 0.994 (0.988-1.001) |  | 0.121 | 0.995 (0.989-1.001) |  | 0.167 | 0.996 (0.991-1.002) |  |
| No | 2213 | 0.672 | 0.998 (0.992-1.005) |  | 0.676 | 0.999 (0.993-1.004) |  | 0.396 | 0.998 (0.993-1.003) |  |

**Abbreviations:** HL, hearing loss; GED, General Educational Development; AA, Associate in Arts; CHF, congestive heart failure; BMI, body mass index; OR, odd ratio; CI, confidence interval.

**Supplementary Table 3. Sensitivity analysis of multivariate logistic regression models assessing the relationship between 25-hydroxyvitamin D2, D3, and hearing loss.**

| **Classifications** |  | | 25(OH)D_2_ | 25(OH)D_3_ |
| --- | --- | --- | --- | --- |
| **Low-frequency HL** | Model 1 | OR ( 95% CI ) | 1.013 (1.005-1.020) | 0.995 (0.991-1.000) |
|  |  | *p*-value | < 0.001 | 0.057 |
|  | Model 2 | OR ( 95% CI ) | 1.012 (1.005-1.020) | 0.996 (0.991-1.001) |
|  |  | *p*-value | 0.001 | 0.167 |
| **Speech-frequency HL** | Model 1 | OR ( 95% CI ) | 1.012 (1.005-1.020) | 0.996 (0.992-1.000) |
|  |  | *p*-value | 0.001 | 0.049 |
|  | Model 2 | OR ( 95% CI ) | 1.010 (1.003-1.018) | 0.997 (0.993-1.001) |
|  |  | *p*-value | 0.006 | 0.177 |
| **High-frequency HL** | Model 1 | OR ( 95% CI ) | 1.003 (0.995-1.011) | 0.996 (0.993-0.999) |
|  |  | *p*-value | 0.438 | 0.023 |
|  | Model 2 | OR ( 95% CI ) | 1.001 (0.994-1.009) | 0.997 (0.994-1.001) |
|  |  | *p*-value | 0.752 | 0.165 |

**Model 1:** adjusted for age, gender, race, education.

**Model 2:** adjusted for age, gender, race, education, BMI, hypertension, diabetes, history of CHF, history of stroke, smoking, supplement use, firearm noise, occupational noise, recreational noise, serum calcium, serum phosphorus, history of renal insufficiency.

**Abbreviations:** 25(OH)D_2_, 25-hydroxyvitamin D_2_; 25(OH)D_3_, 25-hydroxyvitamin D_3_; HL, hearing loss; OR, odd ratio; CI, confidence interval.
